# Induction of Apoptosis by PQ1, a Gap Junction Enhancer that Upregulates Connexin 43 and Activates the MAPK Signaling Pathway in Mammary Carcinoma Cells

**DOI:** 10.3390/ijms17020178

**Published:** 2016-01-29

**Authors:** Stephanie N. Shishido, Thu A. Nguyen

**Affiliations:** Department of Diagnostic Medicine/Pathobiology, Kansas State University, Manhattan, KS 66506, USA; snshishido@gmail.com

**Keywords:** connexin 43, PQ1, gap junction, apoptosis, MAPK

## Abstract

The mechanism of gap junction enhancer (PQ1) induced cytotoxicity is thought to be attributed to the change in connexin 43 (Cx43) expression; therefore, the effects of Cx43 modulation in cell survival were investigated in mammary carcinoma cells (FMC2u) derived from a malignant neoplasm of a female FVB/N-Tg(MMTV-PyVT)634Mul/J (PyVT) transgenic mouse. PQ1 was determined to have an IC_50_ of 6.5 µM in FMC2u cells, while inducing an upregulation in Cx43 expression. The effects of Cx43 modulation in FMC2u cell survival was determined through transfection experiments with Cx43 cDNA, which induced an elevated level of protein expression similar to that seen with PQ1 exposure, or siRNA to silence Cx43 protein expression. Overexpression or silencing of Cx43 led to a reduction or an increase in cell viability, respectively. The mitogen-activated protein kinase (MAPK) family has been implicated in the regulation of cell survival and cell death; therefore, the gap junctional intercellular communication (GJIC)-independent function of PQ1 and Cx43 in the Raf/Mitogen-activated protein kinase/ERK kinase/extracellular-signal-regulated kinase (Raf-MEK-ERK) cascade of cellular survival and p38 MAPK-dependent pathway of apoptosis were explored. PQ1 treatment activated p44/42 MAPK, while the overexpression of Cx43 resulted in a reduced expression. This suggests that PQ1 affects the Raf-MEK-ERK cascade independent of Cx43 upregulation. Both overexpression of Cx43 and PQ1 treatment stimulated an increase in the phosphorylated form of p38-MAPK, reduced levels of the anti-apoptotic protein Bcl-2, and increased the cleavage of pro-caspase-3. Silencing of Cx43 protein expression led to a reduction in the phosphorylation of p38-MAPK and an increase in Bcl-2 expression. The mechanism behind PQ1-induced cytotoxicity in FMC2u mammary carcinoma cells is thought to be attributed to the change in Cx43 expression. Furthermore, PQ1-induced apoptosis through the upregulation of Cx43 may depend on p38 MAPK, highlighting that the effect of PQ1 on gap junctions as well as cellular survival via a MAPK-dependent pathway.

## 1. Introduction

Breast cancer is the most common cancer in women worldwide, and mortality from breast cancer is consistent due to tumor invasion and metastasis [1]. Cell and animal models help to further the understanding of breast cancer pathogenesis and provide insight into treatment options. *In vivo* studies are crucial for drug development, but many drugs do not translate from *in vitro* to *in vivo*. The use of a cell line derived from an animal with clinical features of human breast cancer can be utilized to screen drugs prior to use in the animal itself. Although a number of breast cancer cell lines has been established, a limited number of cell lines is available derived from a transgenic mouse model of spontaneous mammary carcinomas. The establishment of such a line would provide another method to study tumor growth and therapeutic effects despite genetic predisposition for cancer formation. Previously, the transgenic strain FVB/N-Tg(MMTV-PyVT)634Mul/J (also known as PyVT) was used as a model system for measuring tumor burden, drug sensitivity, and metastasis of mammary carcinomas [2]. Here a new cell line, female mammary carcinoma cells (FMC2u), was established from the primary tumor tissue resected from a late stage PyVT mouse with an aggressive, metastatic phenotype. FMC2u was characterized by morphology, receptor expression levels, proliferative and migratory abilities, invasiveness, and colony formation. The anticancer effects on FMC2u cell growth were determined for gap junction enhancers (PQs).

Multiple molecular processes are affected by gap junctional intercellular communication (GJIC), including proliferation, differentiation, migration and apoptosis [3,4]. One of the hallmarks of cancer is the loss of GJIC and connexins, the gap junction proteins [5]. Connexins are therapeutic targets in cancer treatment through both a GJIC-dependent mechanism and a GJIC-independent mechanism [6]. Connexins exhibit a tumor suppressive function via a GJIC-independent manner by interacting and regulating molecules and genes involved in tumorigenesis [7]. The substituted quinolines (PQ1 and PQ7) are gap junction enhancers that have been shown to significantly increase GJIC while reducing breast cancer cell viability [8,9,10]. The inhibitory ability of PQs was tested on FMC2u cellular growth, showing that PQ1 significantly reduced proliferation and viability while increasing Connexin 43 (Cx43) expression. This observation suggests that Cx43 contributes to the anticancer effects of PQ1.

The role of Cx43 in PQ1 induced cytotoxicity was further determined via modulation of expression by transfection experiments in FMC2u cells. Specifically we evaluated the effects of Cx43 modulation on the Raf/Mitogen-activated protein kinase/ERK kinase/extracellular-signal-regulated kinase (Raf-MEK-ERK) cascade and the p38 mitogen-activated protein kinase (MAPK) apoptotic pathway. The MAPKs are signaling pathways critical for the conversion of various extracellular signals to biological responses [11]. In particular, extracellular-signal-regulated kinase (ERK) activation is generally related to cell survival, while reports indicate that apoptosis is associated with the activation of p38 MAPK [11,12,13]. This study explores the role of Cx43 in relation to PQ1 induced cytotoxicity.

## 2. Results

### 2.1. Morphology and Growth Characteristics of Female Mammary Carcinoma Cells (FMC2u)

**Figure 1:**
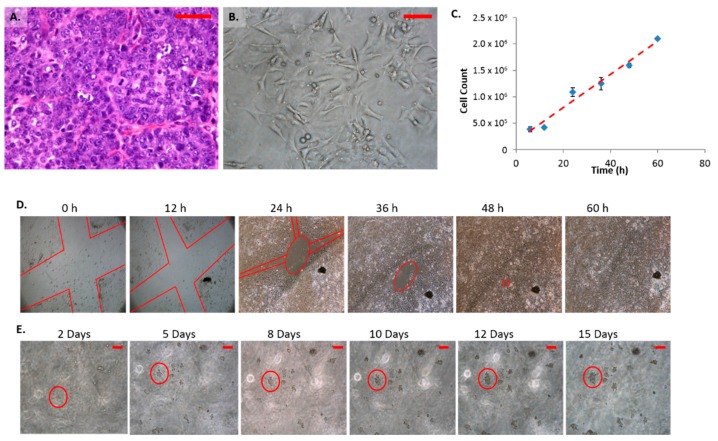
Characteristics of mammary carcinoma (FMC2u) cells. (**A**) H&E of primary tumor isolated from late stage PyVT female at 40×, Scale bar = 50 µm; (**B**) Micrograph of FMC2u in cell culture at 20 Nguyen, ×, Scale bar = 20 µm; (**C**) Proliferation of FMC2u cells with an approximate doubling time of 24 h; (**D**) Migration assay. Red lines indicate a cross section “X” cut in the initial monolayer, approximately 1 mm in diameter. Cells were maintained in RPMI with 10% fetal bovine serum (FBS). Images taken every 12 h at 4×. Wound was completely closed by 60 h; (**E**) Colony formation in soft agar. 10,000 cells seeded on 0.8% agar RPMI and covered with 0.4% agar RPMI. Images were taken every 24 h at 10×. Purple circle follows the growth of a few cells at 48 h to a solid colony at 15 days. Scale bar = 100 µm.

Histologically, the parental tumor was classified as a late carcinoma composed of nests of neoplastic cells that frequently mitosed (Figure 1A). Cells within the tumor contained large pleomorphic nuclei and prominent nucleoli. Characteristic features of the cell line are similar to the parental tumor type and have remained constant despite the number of passages. Cultured FMC2u cells grew as adherent undifferentiated cells (Figure 1B). The nuclei varied in size and little cytoplasm were observed. Cells grew as interlacing colonies with disorganized growth patterns. At heavy cell density the cells were observed to pile upon each other.

Optimal growth conditions were determined via proliferation and viability tests. RPMI media (with or without insulin) with 10% fetal bovine serum (FBS) was found to provide the best conditions for both proliferation and viability of FMC2u (Data not shown). RPMI was used for all following studies. The approximate doubling time was observed to be 24 h (Figure 1C). The growth rate decreased at high cell densities. Invasiveness was determined by the use of transwells with an 8 micron pore membrane. Cells were tested for movement from an insert with 0% FBS to a receiver with 10% FBS RPMI media. Cells were able to transverse the membrane after 24 and 48 h of incubation (Data not shown). Using a “wound” assay FMC2u were observed to migrate efficiently across an adherent surface to form a monolayer of cells (Figure 1D). In 60 h, a 1 mm section was completely closed. Colony formation was determined by use of a soft agar assay in which individual cells were seen proliferating in the agar matrix. Over a 15 day period, large colonies were formed (Figure 1E).

### 2.2. Protein Phenotype of FMC2u Cells

**Figure 2:**
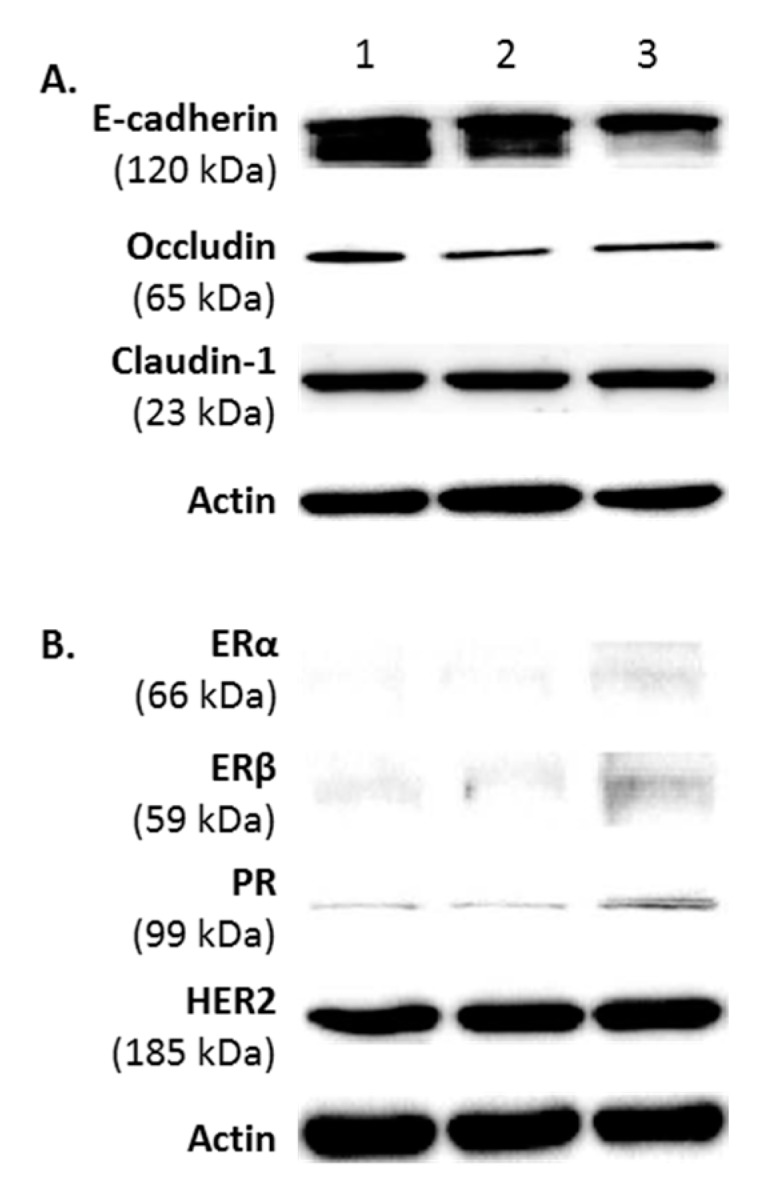

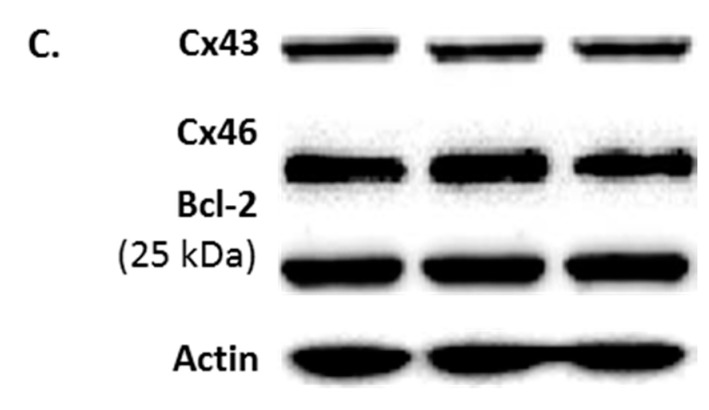
A phenotypic profile of FMC2u cells. Western blot analysis of three different samples of FMC2u (#1–3). (**A**) Images of the epithelial markers *E*-cadherin, occludin, and claudin-1; (**B**) expression of estrogen receptor (ERα and ERβ), progesterone receptor (PR), and human epidermal growth factor receptor 2 (HER2); and (**C**) expression of gap junction proteins (connexin 43 and 46) and molecular marker Bcl-2 are shown using antibodies against specific protein. Actin used as a loading control.

FMC2u cells were determined to be of epithelial origin using the molecular markers *E*-cadherin, occludin, and claudin-1 (Figure 2A). Additionally, the hormone receptor profile was quantified by Western blot analysis which revealed low expression of estrogen receptors α (ERα) and β (ERβ), as well as the progesterone receptor (PR), with strong expression of human epidermal growth factor receptor 2 (HER2) (Figure 2B).

Gap junction intercellular communication (GJIC) has an important function in maintaining tissue homeostasis. GJIC is the process in which small metabolites are shared directly by contiguous cells that have their cytoplasms connected by aqueous channels called gap junctions. FMC2u cells expressed both Cx43 and Cx46 (Figure 2C).

Bcl-2 is an antiapoptotic protein that is a key regulator of apoptosis [14] and associated with low-grade, slowly proliferating, ER+ breast cancer [15,16]. Overexpression of Bcl-2 has been identified in a variety of malignancies and is one of the 21 genes used as a prognostic signature in Oncotype DX, a diagnostic test for the likely benefit from certain types of chemotherapy [17]. FMC2u cells were shown to have a high expression of Bcl-2 (Figure 2C).

### 2.3. Effects of Treatment with Gap Junction Enhancers

A class of substituted quinolines was described in Shi *et al.* [9,18,19] and the effects of the first and second generation compounds (PQ1 and PQ7, respectively) as gap junction enhancers in breast cancer cell lines have been explored in previous studies.

### 2.4. Cellular Proliferation and Viability

**Figure 3:**
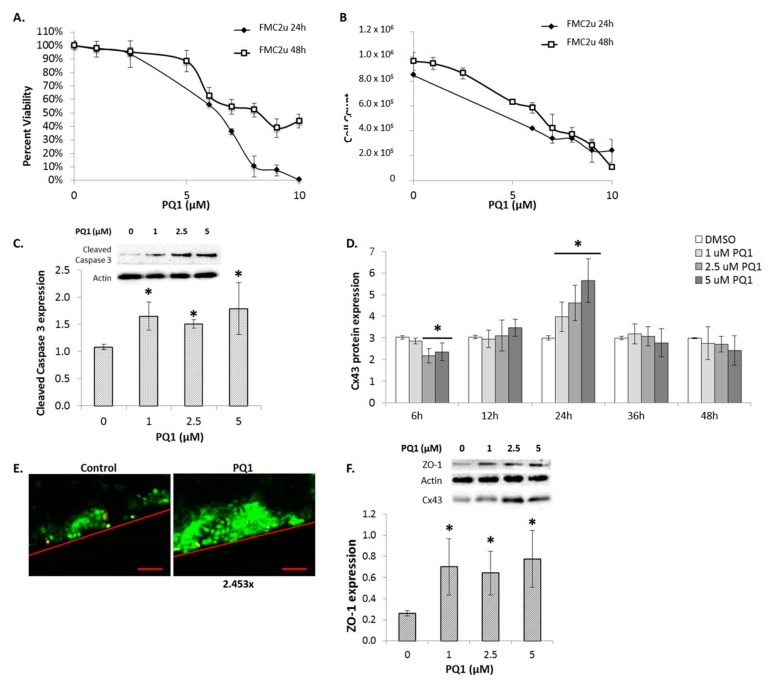
The effects of gap junction enhancer (PQ1) treatment on FMC2u. (**A**) Cellular viability and (**B**) proliferation determined by Acridine Orange/Propidium Iodide (AO/PI) after PQ1 treatment over either 24 or 48 h with varying concentrations; (**C**) Raw and graphical representation of the relative expression of cleaved caspase-3 in FMC2u cells after treatment with 0, 1, 2.5, and 5 µM PQ1 over a 24 h period; (**D**) Graphical representation of Cx43 protein expression in FMC2u cells treated with PQ1 over 6, 12, 24, 36, and 48 h; (**E**) Gap junction activity of FMC2u determined by scrape load dye transfer after treatment with DMSO (control) or PQ1 at 1 µM, for 2 h. Red lines indicate a cross section cut of initial dye. Lucifer yellow was used as a gap junctional dye and Rhodamine-dextran used to mark the cut site. Green fluorescence indicates the passage of dye form the cutting site, showing GJIC. Scale bar = 100 µm; (**F**) Raw and graphical representation of the relative ZO-1 in Cx43-immunoprecipitated complex of FMC2u cells after treatment with 0, 1, 2.5, and 5 µM PQ1 over a 24 h period. Actin used as a loading control. All experiments conducted with a sample size of three. * *p*-value < 0.05 compared to DMSO control.

The gap junction enhancers were tested for their inhibitory capacity on FMC2u cells. PQ1 was shown to have an IC_50_ of 6.5 µM over a 24 h treatment period, while a 48 h treatment period required an increase to 8 µM to reduce viability by 50% (Figure 3A). This suggests that the effect of PQ1 on FMC2u cells is time and dose dependent. The effects of treatment were also seen in the total cell count after each treatment period (Figure 3B), indicating that the proliferative ability of the cells is compromised. PQ7 was shown to be ineffective at all concentrations tested (Data not shown).

To determine if the observed reduction in growth and viability of FMC2u cells after PQ1 treatment was due to activation of the apoptotic pathway, the expression of cleaved caspase-3 was examined. Apoptosis is a highly regulated cell suicide mechanism and the cleavage of protein substrates by caspases is unique to apoptotic cells. PQ1 treatment over a 24 h period led to a significant increase in cleaved caspase-3 expression at all concentrations tested (*p*-value_1µM_ = 0.00556, *p*-value_2.5µM_ = 0.00026, *p*-value_5µM_ = 0.02237; Figure 3C). This indicates that PQ1 activates apoptosis in FMC2u cells.

### 2.5. Connexin Expression

Previous studies have shown that PQ1 may induce an upregulation of Cx43 in neoplastic cells [20,21]. When Cx43-deficient MCF-7 breast cancer cells were transfected with Cx43, the cell-cell communication was restored and their malignant properties including anchorage-independent growth, migration and invasion were reduced, suggesting the role ofCx43 as a tumor suppressor. Immunoblot analysis of Cx43 expression was conducted for FMC2u cells treated with 1, 2.5, and 5 µM PQ1 for 6, 12, 24, 36, and 48 h periods (Figure 3D and Supplemental Figure S1). At 24 h post PQ1 exposure, Cx43 expression is shown to significantly increase in a linear fashion (*p*-value < 0.05). Interestingly, 6 h post treatment, 2.5 and 5 µM PQ1 induced a reduction in Cx43 expression. The anticancer effects of PQ treatment on FMC2u cells may be due to the induced alterations in Cx43 expression.

### 2.6. Gap Junctional Intercellular Communication (GJIC)-Dependent Effect: GJIC and Connexin Protein Binding

To determine functionality of the gap junctions scrape load dye transfer was performed on FMC2u cells with either control (DMSO) or 1 µM PQ1 for 2 h (Figure 3E). The untreated FMC2u cells have functional GJIC, seen from the passage of dye from the cut site to cells further away. PQ1 treatment increased the distance of dye transfer 2.453-fold, indicating an increase in GJIC. The anticancer effects of PQ treatment on FMC2u cells may be due to alterations in Cx43 expression.

Cx43 plays a role in protein binding, specifically to zona occludens-1 (ZO-1). The C-terminal domain of Cx43 contains several phosphorylation sites with signaling motifs which binds to the PDZ domain of ZO-1 [22]. Previous studies showed that endogenous Cx43 and ZO-1 colocalized at the gap junctions [22], suggesting that ZO-1 recruits signaling proteins into the gap junction channel. Immunoprecipitation of Cx43 led to the quantification of bound ZO-1. PQ1 treatment of FMC2u cells resulted in an increase in ZO-1 after 24 h of treatment independent of concentration (*p*-value < 0.05; Figure 3F). The observed increase in bound ZO-1 to Cx43 indicates an increase in signaling molecules recruited to the gap junction. This data indicates that PQ1 has a GJIC-dependent mechanism of action.

### 2.7. GJIC-Independent Effect: MAPK Signaling

**Figure 4:**
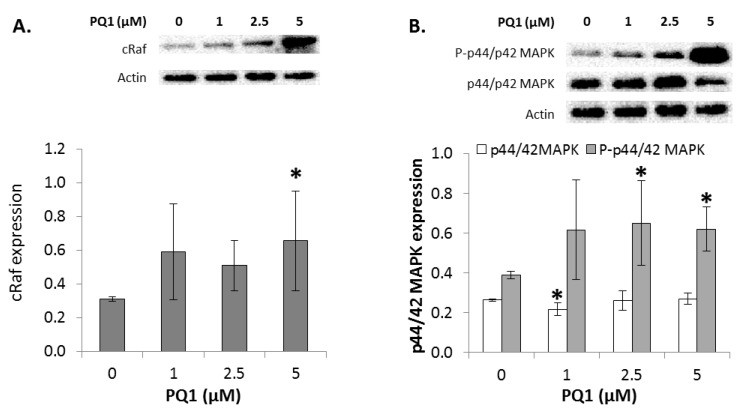

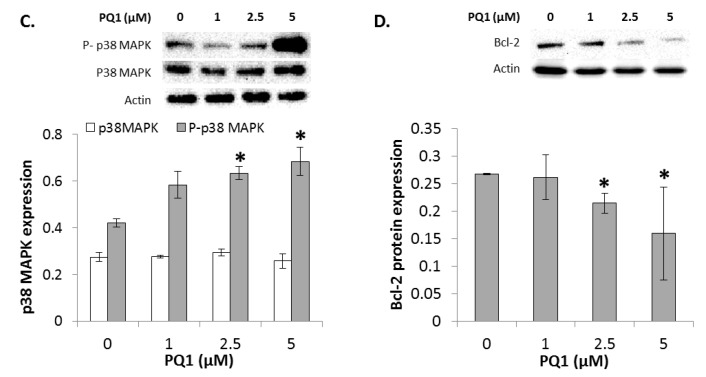
Expression of signaling molecules after treatment with PQ1. Raw and graphical representation of (**A**) cRaf; (**B**) p44/42 MAPK (P: phosphorylated); (**C**) p38 MAPK (P: phosphorylated), and (**D**) Bcl-2 in FMC2u cells after treatment with 0, 1, 2.5, and 5 µM PQ1 over a 24 h period. Actin used as a loading control. All experiments conducted with a sample size of three. * *p*-value < 0.05 compared to DMSO control.

To determine if PQ1 had affected GJIC-independent apoptotic signaling, alterations in the expression of multiple players in the MAPK pathway were identified. Treatment of 5 µM PQ1 induced a significant increase in cRaf expression (Figure 4A). This suggests that PQ1 treatment modulates cRaf expression. Raf kinases are best known as key regulators of the MEK/ERK cascade, and up-regulated signaling through the RAF/MEK/ERK pathway has an important role in cancer [23,24]. Changes in the phosphorylation levels of key proteins in the RAF/MEK/ERK pathway were determined by Western blot analysis. PQ1 treatment over a 24 h period induced an increase in phospho-p44/42-MAPK at 2.5 and 5 µM PQ1 (*p*-value = 0.02606 and 0.00946, respectively; Figure 4B). There were no changes in p44/42-MAPK expression due to PQ1 treatment or transfection experiments. This data indicates PQ1 indirectly alters the ERK1/2 signaling cascade.

Another MAPK family member is the stress-activated protein kinase (p38-MAPK). PQ1 treatment over a 24 h period induced an increase in phosphorylated p38-MAPK when exposed to 2.5 and 5 µM PQ1 (*p*-value = 0.01545 and 0.03686, respectively; Figure 4C). There were no changes in p38-MAPK expression after PQ1 treatment. PQ1, therefore, indirectly alters p38-MAPK signaling cascade, suggesting a MAPK-dependent pathway of apoptosis.

Expression of the antiapoptotic protein Bcl-2 was next determined. This molecular marker is part of a group of antiapoptotic proteins that have been shown to be an important factor in the development of apoptotic resistance. Previous studies have demonstrated that MAPK pathway is active in Bcl-2 overexpressing cancer cells under stressful conditions [25]. Bcl-2 phosphorylation by activated p38-MAPK is a key event in the early induction of apoptosis under conditions of cellular stress [26]. PQ1 has been reported to induce apoptosis in human breast cancer cells through the upregulation of caspases and an alteration in Bax/Bcl-2 expression ratio [21,27]. Exposure to 2 and 5 µM PQ1 induced a significant reduction in Bcl-2 expression in FMC2u cells (*p*-value = 0.00968; Figure 4D). PQ1 treatment affects apoptotic signaling through Bcl-2 expression, possibly due to increased phosphorylation of p38-MAPK.

### 2.8. The Effects of Modulating Connexin 43 Protein Expression

**Figure 5:**
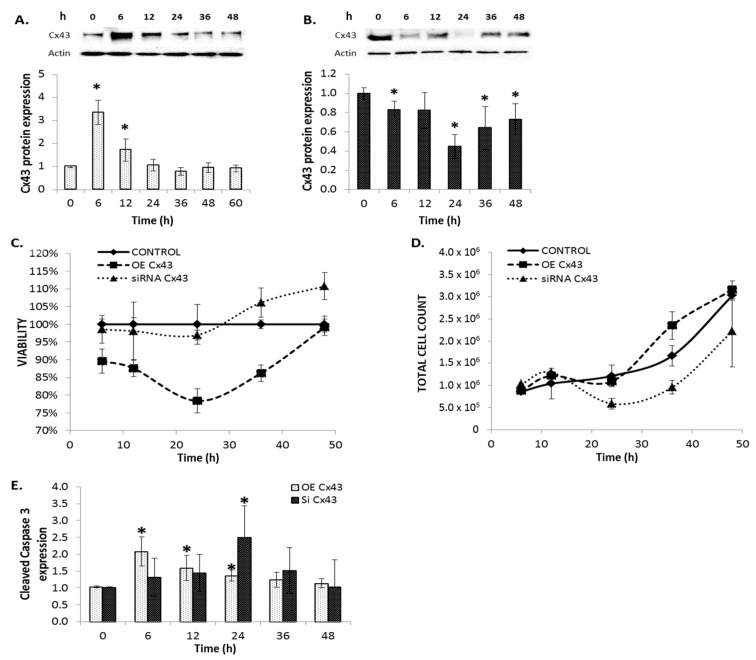
Modulation of connexin 43 expression affects proliferation and viability of FMC2u cells. (**A**) Graphical representation of fold protein induction from western blot analysis of Cx43 in FMC2u cells transfected with GFP-Cx43 cDNA vectors; (**B**) Graphical representation of fold protein induction from western blot analysis of Cx43 in FMC2u cells transfected with siRNA of Cx43; (**C**) Viability and (**D**) Total cell count either overexpressing (OE) Cx43 or with silenced (siRNA) Cx43 compared to normal unmodified cells; (**E**) Graphical representation of cleaved caspase-3 expression in FMC2u cells post transfection. All experiments conducted with a sample size of *n* = 3. * *p*-value < 0.05 compared to respective transfection control.

To determine if the anti-proliferative and apoptotic effects of PQ1 treatment were due to the observed change in Cx43 expression, FMC2u cells were transfected with Cx43 cDNA to induce overexpression. Overexpression of Cx43 was successfully accomplished with an approximate threefold increase in protein expression at 6 h post transfection (Figure 5A). The effects of reduced Cx43 expression were also examined by the transfection of Cx43 siRNA, which successfully silenced approximately 65% of expression at 24 h post transfection (Figure 5B).

### 2.9. Cellular Proliferation and Viability

Overexpression of Cx43 in FMC2u cells had an 86% viability at 6 h post transfection (*p*-value = 0.0037) and remained significantly lower than control cells (Figure 5C). However, silencing of Cx43 by siRNA had a 5% increase in cell viability at 48 h post transfection (*p*-value = 0.0203, Figure 5C). The total cell count was increased at 36 h post-transfection of Cx43 (*p*-value = 0.0150; Figure 5D). Silencing of Cx43 reduced total number of cells at 24 and 36 h (*p*-value_24h_ = 0.0107 and *p*-value_36h_ = 0.0203; Figure 5D). Modulation of Cx43 expression significantly affects the growth and viability of FMC2u cells. 

The expression of cleaved caspase-3, a ubiquitously distributed caspase and the main effector caspase of apoptosis, was determined post transfection of FMC2u cells. Overexpression of Cx43 via transfection of cDNA significantly increased expression of cleaved caspase-3 at 6, 12, and 24 h post transfection (*p*-value_6h_ = 0.0088, *p*-value_12h_ = 0.04275, *p*-value_24h_ = 0.01769; Figure 5E). Silencing of Cx43 significantly increased cleaved caspase-3 expression at 24 h post transfection (*p*-value = 0.035; Figure 5E). Both the overexpression and silencing of Cx43 led to activation of apoptosis in FMC2u cells. This suggests that Cx43 is highly regulated within the cell, and variation in expression levels can trigger cell suicide.

### 2.10. GJIC-Dependent Effect: GJIC and Connexin Protein Binding

**Figure 6:**
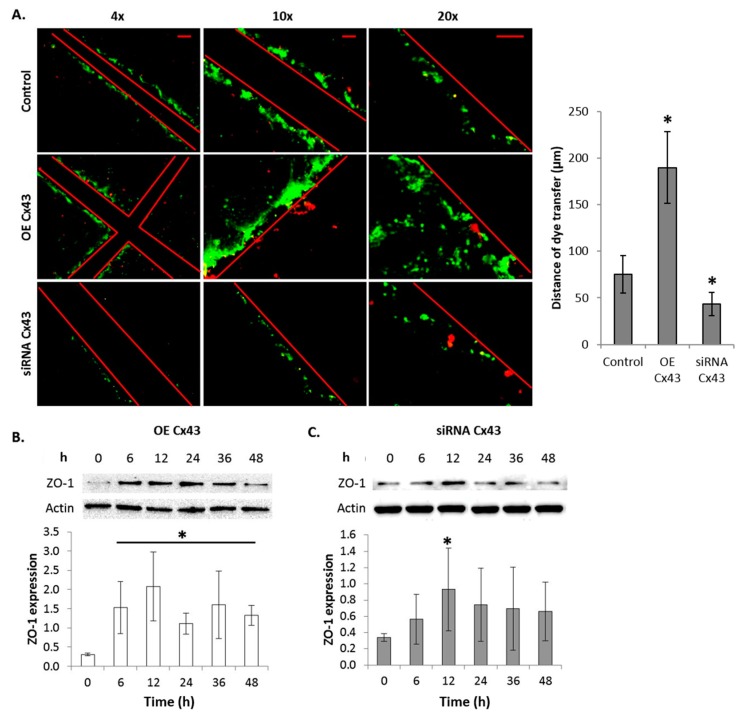
Modulation of connexin 43 expression affects GJIC. (**A**) Gap junction activity of FMC2u determined by scrape load dye transfer after transfection with empty vector (control), GFP-Cx43 cDNA vector, or siRNA Cx43. A graphical representation of dye transfer in µm was shown next to the images. Red lines indicate a cross sectional cut. Lucifer yellow was used as a gap junctional dye and Rhodamine-dextran used to mark the cut site. Green fluorescence indicates the passage of dye form the cutting site. Images taken at 4×, 10×, and 20×. Scale bar = 100, 100, 50 µm, respectively. Graphical representation of ZO-1 expression in Cx43-immunoprecipitated FMC2u cells at 0, 6, 12, 24, 36, and 48 h post transfection; (**B**) Induced overexpression of Cx43 and (**C**) a silencing of Cx43. All experiments conducted with a sample size of *n* = 3. * *p*-value < 0.05 compared to respective transfection control.

To confirm that the overexpression of Cx43 led to functional gap junctions, scrape load dye transfer was conducted. Figure 6A shows an increase in dye transfer in FMC2u cells overexpressing Cx43 compared to control cells. Additionally the silencing of Cx43 resulted in reduced dye transfer in FMC2u cells, indicating a loss of GJIC (Figure 6A). This suggests that exogenous Cx43 assists in the formation of functional gap junctions in FMC2u cells.

Modulation of Cx43 protein expression through transfection experiments induced alterations in ZO-1 expression. Immunoprecipitation of Cx43 led to the quantification of bound ZO-1. Cx43 overexpression led to a significant increase in ZO-1 binding at all experimental time points (Figure 6B). Cells transfected with Cx43 siRNA had little to no effect on ZO-1 expression and binding, only at 12 h post transfection was ZO-1 binding significantly altered (Figure 6C). The observed increase in bound ZO-1 to Cx43 in the overexpressing-Cx43 cells suggests an increase in the recruitment of signaling molecules to the gap junction.

### 2.11. GJIC-Independent: MAPK Signaling

**Figure 7:**
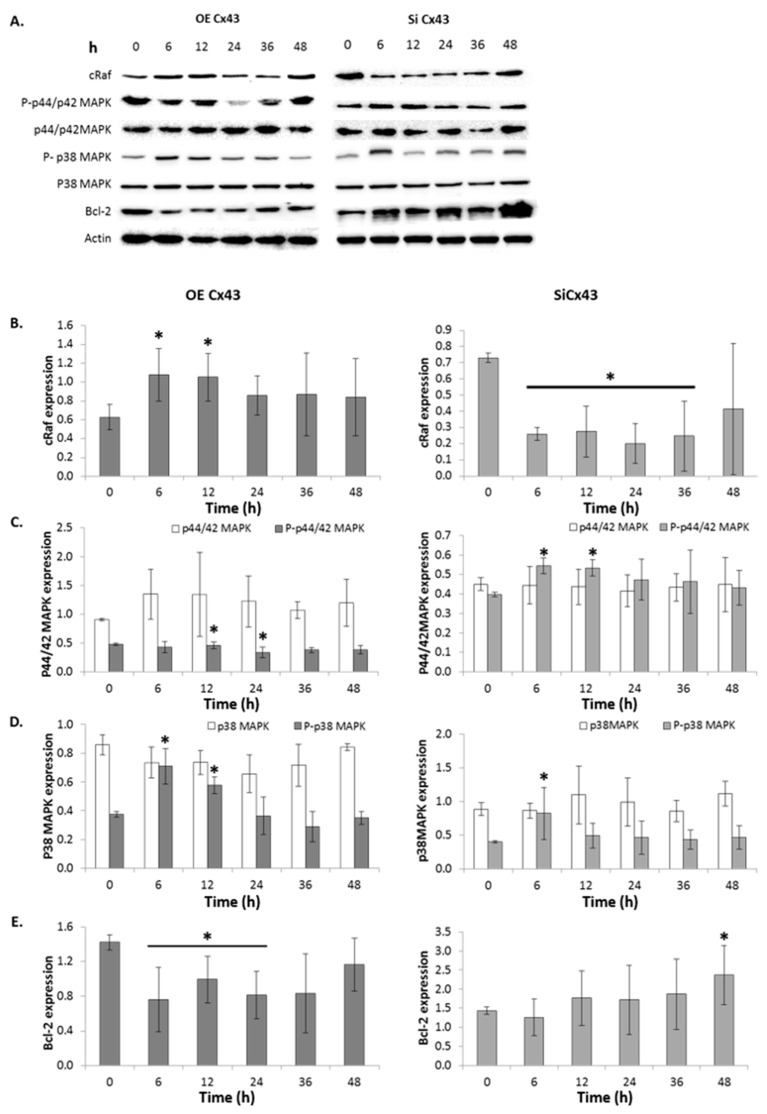
Altering connexin 43 affects expression of signaling molecules involved in cellular survival. (**A**) Raw and (**B**–**E**) graphical representation of western blot analysis of cRaf, phosphorylated (P) and unphosphorylated MAPK (p44/42 and p38), and Bcl-2 expression levels relative to loading control in FMC2u cells transfected with either cDNA of Cx43 to induce overexpression (**Left**) or siRNA to silence Cx43 (**right**). Actin used as a loading control. *n* = 3. * *p*-value < 0.05 compared to respective transfection control.

To determine if Cx43 plays a role in GJIC-independent apoptotic signaling, alterations in the expression of multiple players in the MAPK pathway were identified (Figure 7A). When Cx43 is overexpressed through cDNA, cRaf expression was also significantly upregulated, while silencing Cx43 induced a significant reduction in cRaf expression (Figure 7B). This suggests that Cx43 modulates cRaf expression. 

Overexpression of Cx43 significantly reduced the level of phosphorylated p44/42-MAPK at 24 and 36 h post transfection (*p*-value = 0.04324 and 0.01349, respectively; Figure 7C). With silencing of Cx43 via siRNA there was a significant increase in the phosphorylation of p44/42-MAPK at 6 and 12 h post transfection (*p*-value = 0.00219 and 0.00355, respectively; Figure 7C). There were no changes in p44/42-MAPK expression. This data indicates Cx43 indirectly alters the ERK1/2 signaling cascade, suggesting a role for Cx43 in the regulation of proliferation through ERK1/2 signaling.

Due to overexpression of Cx43 there was a corresponding increase in phosphorylated p38-MAPK expression at 6 and 12 h post transfection (*p*-value =0.00649 and 0.00301, respectively; Figure 7D). Silencing of Cx43 via siRNA led to a reduction in the phosphorylation of p38-MAPK at 6 h post transfection (*p*-value = 0.04942; Figure 7D). There were no changes in p38-MAPK expression due Cx43 silencing. This data indicates Cx43 indirectly alters the p38-MAPK signaling cascade, suggesting a MAPK-dependent pathway of apoptosis.

Overexpression of Cx43 significantly reduced Bcl-2 expression at 6, 12, and 24 h post transfection (*p*-value < 0.05; Figure 7E). Additionally, the silencing Cx43 induced a significant increase in Bcl-2 expression at 48 h post transfection (*p*-value = 0.03119; Figure 7E). This indicates that altering Cx43 expression affects apoptotic signaling through Bcl-2 expression, most likely due to increased phosphorylation of p38-MAPK.

## 3. Discussion

In this study, the female mammary carcinoma cell line FMC2u, derived from a MMTV-PyVT transgenic mouse, was established and characterized over multiple passages. FMC2u cells are adherent cells of epithelial origin that proliferate rapidly. Growth characteristics and protein analysis suggests that these cells represent an advanced stage of mammary carcinoma in a mouse model. FMC2u cells have been shown to both migrate across an adherent surface and transverse a membrane with 8 micron pores, indicating a more invasive neoplastic cell line. FMC2u cells are highly proliferative with a doubling time of 24 h. This is a rapidly growing cell line with greater proliferative ability than MCF-7, T47D, MDA-MB-453, and MDA-MB-231 breast cancer cell lines, which double at 29, 36, 47, and 38 h respectively. The more proliferative the cell line, the more rapidly the tumor grows, and a tendency to have a poorer prognosis.

Hormone receptor profiles are useful diagnostic tools and predictors of therapeutic response. FMC2u were determined to express low levels of ER and PR, with high expression of HER2. These cells can be considered HER2 + high/ER + low/PR + low. ER is expressed in approximately 80% of all breast cancers [28], while overexpression of HER2 or both are present in about 15% of breast cancers [29]. HER2 overexpression is associated with partial resistance to endocrine treatment [30,31], suggesting that this cell line would be unresponsive to endocrine therapy. Additionally, RAS signaling pathways are commonly activated in tumors in which growth-factor-receptor tyrosine kinases, such as HER2, have been overexpressed. Aberrant overexpression or mutational activation of receptor tyrosine kinases can cause hyperactivation of Ras leading to upregulated MAPK signaling [32,33,34]. The overexpression of HER2 may enhance the MAPK signaling cascade, making this cell line appropriate for MAPK specific research.

Impaired gap junctional intercellular communication (GJIC) has been reported *in vivo* in many human tumors [35,36] and *in vitro* in response to oncogenes [37] or tumor promoters [38]. Primary tumors that are initially GJIC impaired become GJIC competent during the metastatic stage [4]. Increased expression of connexins and GJIC correlate with invasiveness and metastasis in a variety of cancer cell types, including breast cancer. Connexin expression profiles change from a metastatic cell to that more similar to a normal breast epithelial cell with expression of metastasis-suppressor gene BRMS1 [39]. This suggests that the connexin composition of gap junctions contributes to the lesions metastatic potential. FMC2u cells were shown to be GJIC competent with strong expression of Cx43. Previous data presented suggests that Cx43 and Cx46 are upregulated during late tumor development and metastasis in the parental transgenic mouse model [20]. The report also demonstrated that expression of HER2 at the three stages of tumor development is higher in the Early and Late stages than the Pre stage. Furthermore, examination of connexins in 96 breast cancer patients showed that pre-chemotherapy Cx43 expression correlated positively with hormone receptor status both before and after chemotherapy and had a negative correlation with HER2 expression pre-chemotherapy [40]. These findings suggest that there is a negative correlation between Cx43 and HER2 expression.

PQ1 was shown to have an IC_50_ of 6.5 µM over a 24 h treatment period. This concentration is 6.5 times that needed to inhibit T47D growth [9], indicating the FMC2u are more resistant to treatment than other cells lines tested. PQ1 is considered a gap junction enhancer due to the observed increase in GJIC and upregulation of connexin expression [9,20,21]. Here, PQ1 treatment of FMC2u cells resulted in the upregulation of Cx43, with a decrease in cellular viability and proliferation rate. This may be explained by the overexpression of Cx43 induced by PQ1. The role of Cx43 both as a gap junctional component and independent of GJIC was examined in FMC2u cells with the goal of providing insight into the mechanism behind the anticancer effects of PQ1.

Overexpression by transfection of several connexins, such as Cx43 in tumor and transformed cells, has been shown to inhibit tumorigenicity, leading to the notion that connexins behave like tumor suppressor genes and may be involved in physiological and/or tumor cell growth [41,42] given the critical role of Cx43 expression. Transfection experiments conducted indicate that a reduction in connexin expression slows neoplastic cell proliferation, while an increase in connexin expression reduces viability. This suggests that there are multiple pathways affected by alterations in Cx43 expression.

Connexins can interact with a large number of scaffolding and signaling proteins that are involved in the regulation of GJIC [43,44]. ZO-1 is a major connexin interacting protein needed for stability of Cx43 at the plasma membrane and endocytosis of the gap junction [45,46,47]. Disruption of ZO-1-Cx43 interaction in osteosarcoma cells has been shown to lead to a decreased in GJIC and delocalized Cx43, while overexpression of ZO-1 increases GJIC and membranous Cx43 [48]. The dissociation of ZO-1 from Cx43 results in gap junction endocytosis, indicating that Cx43 internalization is a ZO-1 dependent process [49]. Here, we show that ZO-1 is bound to Cx43, indicating functional gap junction plaques in FMC2u cells. The observed increase in ZO-1 with Cx43 overexpression suggests an increase in recruitment of signaling molecules towards the gap junction and thus an increase in GJIC. This indicates that the upregulation of connexin expression by PQ1 treatment leads to an increase in ZO-1 binding and GJIC. PQ1 has a GJIC-dependent mechanism of action.

Connexins have been shown to be tumor suppressors, but rather than being dependent on cell coupling activity, this effect has also been linked to connexin species-specificity [41,50]. Connexin-transfected cells with inhibition of cellular growth and tumor suppression lack gap junction assembly on the cell membrane [51] and have cytoplasmic and nuclear localized connexin expression [41,52], indicating gap junctions and hemichannels are not involved. Additionally, only the c-terminal domain of specifically Cx43 has been shown to be vital for growth suppression [53,54]. These data suggest that expression of connexins, rather than GJIC, corresponds to the regulation of neoplastic growth and suppression. Further study will be explored into the GJIC-independent role of Cx43 in relation to PQ1 cytotoxicity in FMC2u cells.

The mitogen-activated protein kinase (MAPK) signaling pathways are crucial for the maintenance of the cells. There are three subfamilies of MAPKs: extracellular signal-regulated kinases (ERKs, p44/42 MAPK), c-Jun N-terminal kinases (JNKs), and p38-MAPKs. In general, ERKs are important for cellular proliferation and survival, while JNKs and p38-MAPKs respond to stress stimuli to induce apoptosis [55]. First we observed the expression of cRaf, a protooncogene product that is a main component of many signaling pathways involved in normal cellular proliferation and oncogenic transformation [56]. Upon activation, cRaf phosphorylates the mitogen-activated protein kinase kinase (MEK), which then can activate p44/42 MAPK which propagates the signals [56]. PQ1 treatment led to an upregulation of cRaf expression and a corresponding increase in the activation of p44/42 MAPK, indicating an increase in the ERK1/2 signaling cascade. Unexpectedly, the overexpression of Cx43 led to a different result, in which there was an upregulation of cRaf, but a reduction in activation of p44/42 MAPK. This suggests that PQ1 has a Cx43-independent effect on the ERK1/2 signaling cascade.

Transfection of Cx43 siRNA induced a reduction in GJIC while leading to a decrease in cRaf expression, but an activation of p44/42 MAPK. This is the opposite of what was seen in overexpression experiments, indicating that Cx43 may play a role in the regulation of ERK1/2 signaling, and thus cellular growth and survival. This also supports the hypothesis that cells must be removed from a state of growth suppression prior to mitogenic cell signaling [57]. The inhibition of GJIC has previously been shown to precede the activation of p44/42 MAPK [58]. This is confirmed by the observation that reduced expression of Cx43 induced a decrease in GJIC with a corresponding activation of ERK signaling. The Raf-MEK-ERK cascade is a core element of a complex signaling network. More research is needed to determine how Cx43 may contribute to the Raf-MEK-ERK pathway in cancer.

Recent reports have shown the PQ1 has anticancer properties as a potent chemopreventive and chemotherapeutic compound that prevents, inhibits, and attenuates tumorigenesis [20,21,27]. The results of the present study are in agreement with the previous studies concerning activation of apoptotic signaling with PQ1 treatment. The cytotoxicity of PQ1 may be attributed to the upregulation of Cx43. PQ1 also led to the phosphorylation of p38-MAPK. This suggests activation of apoptosis, which is confirmed by the cleavage of caspase-3 and reduced cellular viability. There was a corresponding reduction in Bcl-2, indicating the cell is more susceptible to apoptotic signaling. These results match those from transfection experiments in which Cx43 is overexpressed. Therefore, PQ1 induces apoptosis via modulation of Cx43, resulting in direct effects on tumor cell survival in a p38 MAPK-dependent manner.

The effects of treatment with the gap junction enhancer PQ1 reduced proliferation and viability, while also inducing an upregulation in Cx43 expression in an epithelial based mammary carcinoma cell culture derived from a malignant murine tumor. PQ1 was shown to not only affect GJIC, but also cellular survival via a MAPK-dependent pathway. The activation of p44/42 MAPK by PQ1 was independent of Cx43 expression, but modulation of Cx43 was shown to alter ERK signaling. This introduces the hypothesis that Cx43 may contribute to the regulation of the ERK1/2 signaling cascade. Additionally, Cx43 may be the key element in the mechanism of PQ1 induced apoptosis. The present study provided the first evidence that PQ1 activates p38 MAPK in an aggressive mammary carcinoma cell line.

Further studies will analyze the effect of Cx43 siRNA and overexpression on PQ1-induced molecular effects, GJIC, and apoptosis. Initial finding of mechanistic link demonstrates that 5 µM PQ1 can increase Cx43 expression in prior treated cells with Cx43 siRNA (Supplemental Figure S2). Further analysis is needed to fully examine the effect of PQ1-induced apoptosis.

## 4. Experimental Section

### 4.1. Ethics Statement

Husbandry of animals is conducted by the Comparative Medical Group (CMG) at the College of Veterinary Medicine at Kansas State University (Manhattan, KS, USA). The CMG animal facilities are fully accredited by the Association for Assessment and Accreditation of Laboratory Animal Care, International (AAALAC). The compliance to aspects of animal welfare law is regularly monitored by the veterinary staff. Animal care and use protocols were approved by the Institutional Animal Care and Use Committee (IACUC) at Kansas State University, Manhattan following NIH guidelines.

### 4.2. Establishment of Cell Cultures

FMC2u cultures were derived from malignant tumors from the transgenic strain FVB/N-Tg(MMTV-PyVT)634Mul/J. Female mice were approximately 12 weeks old with no treatment prior to tissue removal. Whole tumors were washed with sterile PBS three times and cut in half prior to trypsinization for 30 min at 37 °C. After incubation, tumors were removed and trypsin was neutralized with RPMI with insulin, followed by centrifugation at 2000 rpm for 5 min. Supernatant was aspirated and cells resuspended in RPMI supplemented with insulin and 10% fetal bovine serum (FBS) and then plated in a T-75 tissue culture flask. Cells were maintained in RPMI medium supplemented with 10% FBS (Atlanta Biologicals, Lawrenceville, GA, USA) at 37 °C with 5% CO_2_ in T-125 flasks.

### 4.3. Media Test

Cells were seeded in 6-well plates in RPMI with insulin, RPMI, DMEM, DMEM/F12, MEM, or L15. All media contained 10% FBS. Images were taken of the monolayer growth every 24 h. Once cells were 100% confluent, cell count and viability were determined by AO/PI (see below).

### 4.4. Cell Doubling Time

Cell doubling times at 36 °C were measured by counting the number of viable cells from trypsinized monolayers by Trypan blue exclusion. Counts were performed at 12 to 24 h intervals.

### 4.5. Colony Formation

100,000 cells were seeded between 0.8% agarose RPMI 10% FBS and 0.4% agarose RPMI 10% FBS. Images were taken every 24 h to determine if individual cells could form a colony in a 3 dimensional medium.

### 4.6. Migration Assay

Cells were grown to 90% confluency. Two cuts crossing each other in the center of the well were made to create a “wound” on the cell monolayer. Cells were washed with 1 mL of media and then incubated in RPMI 10% FBS at 37 °C. Images were taken every 12 h until the wound was closed and cells were confluent.

### 4.7. Invasion Assay

Cells were seeded into the upper chamber of a transwell with RPMI 0% FBS. The lower chamber contained RMPI 10% FBS. Cells were incubated for varying time points (24, 48, 72 h) and the number of cells in the lower chamber were counted by Trypan blue.

### 4.8. Cell Recovery

Cells were frozen down using 500 µL of cell culture freezing medium from Invitrogen (Cat. # 12648-010; Life Technologies, Grand Island, NY, USA) and allowed to sit at −20 °C for 1 h prior to exposure to −80 °C. Cryovials were removed from −80 °C storage and rapidly thawed with warm media. Cells were gently agitated. Samples were transferred to 15 mL conical tubes and 5 mL of warm media was added. Cells were allowed to incubate for 10 min at 37 °C, followed by centrifugation (5 min at 2000 rpm). Supernatant was aspirated out and cells resuspended in PBS and Trypan Blue for viability testing.

### 4.9. Antibodies

Antibodies against cleaved caspase 3, Cx43, Cx46, ER (α and β), PR, and Bcl-2 (Santa Cruz Biotechnologies, Santa Cruz, CA, USA), phospho-p42/44 MAP kinase, cRaf, and HER2 (Cell Signaling, Boston, MA, USA), and actin (Sigma-Aldrich, St. Louis, MO, USA) were used.

### 4.10. Western Blot Assay

Whole cell extractions conducted using lysis buffer (20 mM Tris pH 7.5, 0.5 mM EDTA, 0.5 mM EGTA, and 0.5% Triton X-100) with 1:1000 dilution of protease inhibitors (Sigma-Aldrich, Saint Louis, MO, USA). Tissue was sonicated, followed by centrifugation at 13,000 rpm for 30 min at 4 °C. Twenty-five μg of whole-cell extract was resolved by 10% SDS polyacrylamide gel electrophoresis (PAGE) and transferred to nitrocellulose membrane (Midwest Scientific, Saint Louis, MO, USA). Nitrocellulose membrane was blocked in 5% milk for an hour at room temperature and then incubated with monoclonal antibodies at a dilution of 1:1000. Western blots were detected by enhanced chemiluminescence detection reagents (Pierce, Rockford, IL, USA) and visualized by Fluorochem E imaging system (ProteinSimple, San Jose, CA, USA).

### 4.11. Immunoprecipitation Assay

For immunoprecipitation 1 mg of total protein was precleared with 10 µL of IgG and 10 µL protein A/G agarose beads (sc-2003, Santa Cruz Biotechnologies, Santa Cruz, CA, USA). Samples were incubated at 4 °C for 30 min, followed by centrifugation at 1500 rpm for 5 min to pellet the IgG agarose conjugate. Five microliters of primary antibody were added to each sample and incubated at 4 °C overnight. This was followed by addition of 20 µL protein A/G agarose beads and 2 h incubation. Sample was centrifuged at 1500 rpm for 5 min to pellet the primary antibody agarose conjugate. Supernatant was removed and remaining pellet used for Western blot analysis.

### 4.12. Treatment with Gap Junction Enhancers

Compound PQ1, 6-methoxy-8-[(3-aminopropyl)amino]-4-methyl-5-(3-trifluoromethyl-phenyloxy)quinolines, was prepared by following the reported procedure [18]. Cells were seeded into 6-well plates in RPMI 10% FBS and allowed to attach overnight. Cells dosed with varying concentrations of each compound in RPMI 0% FBS for 24, 48, or 72 h.

### 4.13. Overexpression and Silencing of Connexins

cDNA from human Cx43 was purchased from Addgene (Cambridge, MA, USA; plasmid #40907). Connexin 43 siRNA (sc-35091) was purchased from Santa Cruz Biotechnolgoy (Santa Cruz, CA, USA). Connexin 46 siRNA (S100131670) was purchased from Qiagen (Germantown, MD, USA). DNA and siRNAs were transfected according to the manufacturer’s specifications using Polyplus (Illkirch, France) jetPEI-FluoR (106-05N) and Mirus (Madison, WI, USA) *Trans*IT-siQUEST (MIR 2114) transfection reagent, respectively. Briefly, cells were cultured in six-well plates and incubated under their normal growth conditions. When cells reached 50% confluency, 5 μg of DNA or 3.2 μg of siRNA complex was mixed with transfection reagent and incubated at room temperature for 15 min prior to applying to the cells. Cells were either utilized for *in vitro* experimentation or the cell lysates were collected and prepared for Western blot analysis.

### 4.14. Proliferation and Viability

Post treatment period, the media was removed and saved. The cells were trypsinized for 5 min at 37 °C. Cells were scraped off the plate and transferred to the saved media. Wells were washed with 3 mL of PBS and elution was saved. Media, cells, wash were centrifuged for 4 min at 2000 rpm. Supernatant was aspirated and cells resuspended in PBS. A cell sample was mixed with Acridine Orange/Propidium Iodide (AO/PI) at a 1:1 ratio. Cell count and viability was determined by Nexcelom (Lawrence, MA, USA) cell counter.

### 4.15. Scrape Load Dye Transfer

Cells were grown to 90% confluency on coverslips in 6-well plates. Cells were washed three times with PBS. The 2.5 μL mixture of 1% (*w*/*v*) Lucifer yellow and 0.75% (*w*/*v*) of Rhodamine-dextran was added in the center of the coverslip. Two cuts crossing each other in the center of the coverslip were made. After 3 min, cells were washed three times with PBS and incubated at 37 °C in tissue culture media for 20 min. The cells were then washed with PBS and fixed in 2.5% paraformaldehyde for 10 min. Cells were mounted on a slide, sealed and visualized under a fluorescence microscope at 10× objective. Three independent experiments with sample size of 3 per each treatment were performed.

### 4.16. Statistical Analysis

Significance was considered at a *p*-value ≤0.05 using Student’s *t*-test analysis. All data are presented as mean ±95% confidence interval of at least three independent experiments.

## 5. Conclusions

A novel cell line, FMC2u, from the primary tumor with an aggressive, metastatic phenotype was used to evaluate the role of Cx43 in PQ1-induced cytotoxicity. The findings show that PQ1 decreased cell viability and proliferation and induced upregulation of Cx43. Furthermore, PQ1 exerts GJIC-independent effects by modulating ERK1/2 and p38-MAPK signaling and by decreasing the expression of Bcl-2 and increasing the cleavage of pro-caspase-3. However, overexpression of Cx43 resulted in reduced expression of p44/MAPK, while PQ1 treatment had the opposite effect, which suggests that PQ1 affects the Raf-MEK-ERK cascade independent of Cx43 upregulation. Therefore, the mechanism behind PQ1-induced cytotoxicity in FMC2u is likely due to the change in Cx43 expression. PQ1-induced apoptosis through the upregulation of Cx43 may depend on p38 MAPK, highlighting that the effect of PQ1 on gap junctions as well as cellular survival via a MAPK-dependent pathway.

## Supplementary Materials

The following are available online at www.mdpi.com/2073-4360/8/2/48/s1.
