# Supplementary material for: Induction of Apoptosis by PQ1, a Gap Junction Enhancer that Upregulates Connexin 43 and Activates the MAPK Signaling Pathway in Mammary Carcinoma Cells
Source: Int J Mol Sci. 2016 Jan 29;17(2):178. doi: 10.3390/ijms17020178 (PMC4783912; doi:10.3390/ijms17020178)
Supplement: Supplementary file 1 [file ijms-17-00178-s001.pdf]

Supplementary Materials: Induction of Apoptosis by PQ1, a Gap Junction Enhancer that Upregulates Connexin 43 and Activates the MAPK Signaling Pathway in Mammary Carcinoma Cells

Stephanie N. Shishido and Thu A. Nguyen

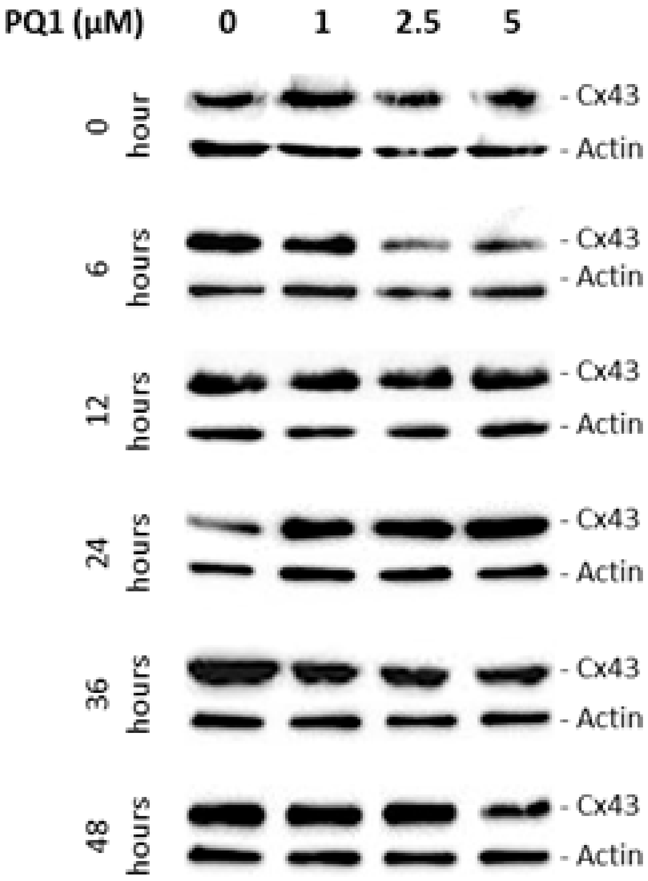

Supplemental Figure 1. Western blot analysis of Cx43 in FMC2u cells treated with various concertratrons of PQ1 over 6, 12, 24, 36, and 48 h. Actin was used as a loading control.

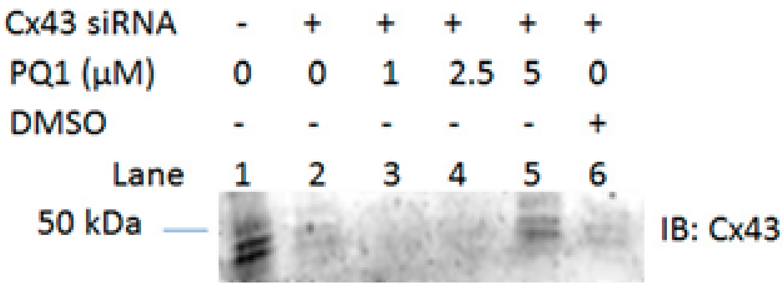

Supplemental Figure 2. Expression of Cx43 in cells transfected Cx43siRNA and dosed with various concentrations of PQ1 for 24 h.
